# Exploring App-Based Physiotherapy for Somatic Tinnitus: Results from a Pilot Study

**DOI:** 10.3390/jcm13237203

**Published:** 2024-11-27

**Authors:** Sarah Michiels, Stella Wölflick, Jorge Simões, Winfried Schlee

**Affiliations:** 1 REVAL Rehabilitation Research Center, Faculty of Rehabilitation Sciences, Hasselt University, 3590 Diepenbeek, Belgium; 2 Department of Otorhinolaryngology, Antwerp University Hospital, 2000 Edegem, Belgium; 3 Klinik und Poliklinik für Psychiatrie und Psychotherapie der Universität Regensburg am Bezirksklinikum Regensburg, Universität Regensburg, 93053 Regensburg, Germany; 4 Department of Health Psychology and Technology, University of Twente, 7500 AE Enschede, The Netherlands; 5 Institute for Information and Process Management, Eastern Switzerland University of Applied Sciences, 9001 St. Gallen, Switzerland

**Keywords:** tinnitus, somatic tinnitus, physical therapy, smartphone applications

## Abstract

**Background:** Somatic tinnitus (ST) is a type of tinnitus that is influenced by changes in somatosensory input from the cervical spine or temporomandibular area. Although traditional physiotherapy has been shown to reduce ST symptoms, in-clinic treatment is not always available, and adherence to home exercise programs is often low. This study aims to investigate the effectiveness of an app-based physiotherapy program to enhance the compliance and availability of treatment for ST patients. **Methods:** This pilot randomised controlled trial included 38 adult patients with chronic somatic tinnitus. Participants were randomly assigned to a treatment group receiving a 9-week app-based cervical spine exercise program or a control group. The primary outcome was tinnitus distress measured using the Tinnitus Handicap Inventory (THI). As a secondary outcome we used the Mini-Tinnitus Questionnaire (Mini-TQ). **Results:** Participants in the treatment group showed significant reductions in tinnitus distress, with a large effect size for both the THI and Mini-TQ groups (Cohen’s d = 1.71 and 1.02, respectively). The control group showed no significant changes. **Conclusions:** This study provides evidence that an app-based physiotherapy intervention is a feasible and effective treatment for ST. Further research with larger sample sizes and comparisons to traditional in-clinic treatments is needed to confirm these findings. Additional studies on personalised treatment might further improve the treatment.

## 1. Introduction

Tinnitus is the perception of sound in the absence of an external source for the sound. It occurs in 10–15% of adults [1] and strongly interferes with daily functioning and quality of life in 5–10% of patients [2]. Tinnitus is often related to hearing loss or noise trauma, but it can also be related to changes in somatosensory input from the cervical spine or temporomandibular area, which is called somatosensory or somatic tinnitus (ST) [3,4].

A physiological explanation for ST has been found in the existence of connections between the somatosensory system of the cervical spine and temporomandibular area on the one hand and the cochlear nuclei on the other hand [5]. Cervical and temporomandibular somatosensory information is conveyed to the brain by afferent fibres, the cell bodies of which are located in the dorsal root ganglia or the trigeminal ganglion. Some of these fibres also project to the central auditory system [6]. These projections enable the somatosensory system to influence the auditory system by altering the spontaneous firing rates or synchronicity of firing among neurons in the cochlear nucleus, inferior colliculus, or auditory cortex [6,7]. In this way, altered afferent information caused by muscle tension or decreased mobility can change the pitch or loudness of an existing tinnitus perception or can even cause tinnitus. Muscles in the TMJ area and the neck have been related with changes in tinnitus perception. Recent evidence has demonstrated a relationship between the temporomandibular joint (TMJ) and the cervical region, showing that cervical myofascial pain is linked to an imbalance in masticatory muscle activity [8].

Currently, most treatment options for ST are focused on normalising the afferent input from the cervical spine and temporomandibular area [9]. This would then result in a decrease in tinnitus loudness via the same connecting fibres described above. A multimodal physiotherapy treatment consisting of a combination of manual mobilisations by a physiotherapist and exercises that are mostly performed at home has been shown to provide extensive tinnitus relief in patients with ST [9,10,11,12,13,14,15]. The exercise part of the treatment is essential to avoid therapist dependence and stimulate patients’ self-efficacy. However, previous research has shown that up to two-thirds of patients are not compliant in performing daily home exercises [16], and others experience difficulties in performing the exercises correctly without the guidance of the physiotherapist. As such, this group would benefit from additional support to increase exercise compliance.

In recent years, computer and smartphone applications have been increasingly finding their way into medicine and physiotherapy treatment. The COVID-19 pandemic has recently shown that parts of treatment and counselling can be provided from a distance through applications and videoconferencing instead of (or in combination with) face-to-face clinical care. Telerehabilitation and internet-based therapy have already proven their effectiveness in various musculoskeletal conditions [16] and tinnitus [17], respectively. As such, the care for ST patients would also benefit from digitalisation. The use of smartphone applications for tinnitus has become more prevalent in recent years [18,19]. A specific smartphone application for the treatment of ST, however, has not been studied yet. Therefore, this study aims to investigate the effectiveness of a smartphone application specifically directed at treating patients with ST to test the hypothesis that 9 weeks of app-based treatment of ST will lead to a decrease in tinnitus symptoms.

## 2. Materials and Methods

### 2.1. Design

**Figure 1:**
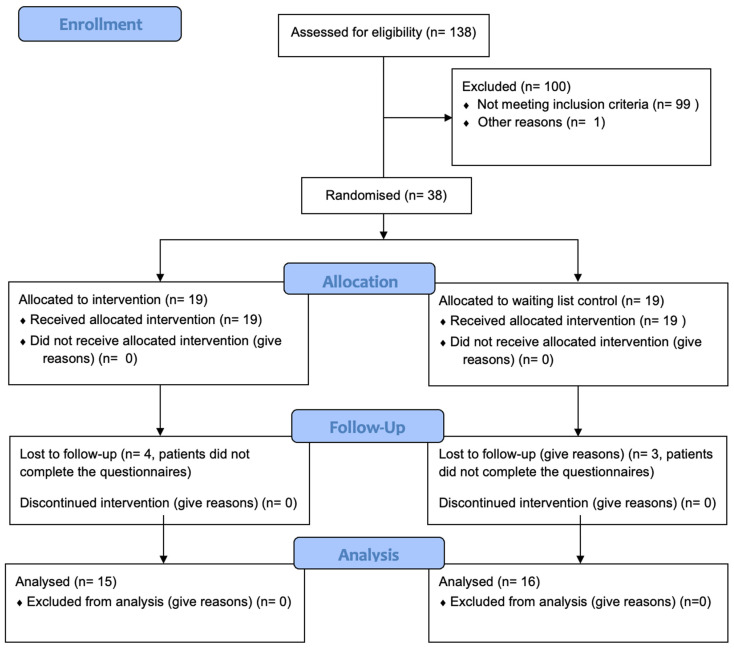
Study flowchart. THI: Tinnitus Handicap Inventory; ESIT-SQ: European School for Inter disciplinary Tinnitus Research Screening Questionnaire; STQ-SF: Somatic tinnitus questionnaire short form.

The study was designed as a two-arm randomised controlled trial. After screening for eligibility, patients were randomised to either the treatment or the control group in a 1:1 ratio based on block randomisation with variable block length. A concealed randomisation list was generated using random numbers. A stratified randomisation according to the Tinnitus Handicap Inventory (THI) score was used. Based on the THI score during screening, patients were divided into a ‘low tinnitus distress group’ (THI < 43) and a ‘high tinnitus distress group’ (THI ≥ 43). The stratified randomisation assured an even distribution of patients with ‘low’ and ‘high’ tinnitus distress in both study arms. An overview of the study design is presented in Figure 1. The pilot study was registered at the German Register for clinical studies (DRKS registration number: DRKS00031225) and was approved by the Ethics Committee of the University Hospital Regensburg (registration number 21-2230-101, approval date: 24 February 2021). The data were collected between August 2021 and November 2022.

### 2.2. Participants

Participants were recruited via the Multidisciplinary Tinnitus Clinic at the University of Regensburg and its communication channels on social media, the press, and information from the medical staff. The demographic data and clinical characteristics of tinnitus, which may influence the study results, were registered. Therefore, the European School for Interdisciplinary Tinnitus Research Screening Questionnaire (ESIT-SQ), including 39 questions about risk factors for tinnitus and tinnitus characteristics, was used [20]. Data from the ESIT-SQ were collected during screening. Participants in the control group received no treatment during their study participation but were offered the experimental treatment after completing the last study measurement.

### 2.3. Inclusion and Exclusion Criteria

Adult patients (+18 years old) with chronic tinnitus (≥six months) were included in the study when diagnosed with somatosensory tinnitus (ST) based on the Somatic Tinnitus Questionnaire—Short form (STQ-SF) [21]. Apart from being diagnosed with ST, patients’ tinnitus distress had to be mild according to the THI score (≥18 points). Patients had to be fluent in English to complete the treatment. Additionally, all included patients had to own a smartphone (iOS or Android) and had to be able to use the most common applications without support. Patients with otological or neurological conditions such as otosclerosis, acoustic neuroma, or Meniere’s disease were excluded from participation in the study.

### 2.4. Intervention

The treatment group received a 9-week app-based cervical spine exercise program that was based on exercises used in previous studies investigating in-clinic physiotherapy treatment [10,14]. The program consisted of exercises to increase strength, endurance, and coordination of the cervical spine (deep neck flexor and extensor muscles) and shoulder stabilising muscles and exercises to increase mobility and improve posture [10,13]. In total, 16 different exercises were included in the program at 5 different difficulty levels to build a 9-week program with increasing difficulty over time. Each week had the same three exercises for the entire week, and the difficulty was increased throughout the program by increasing the number of repetitions of the same exercise or moving on to a more difficult exercise.

**Figure 2:**
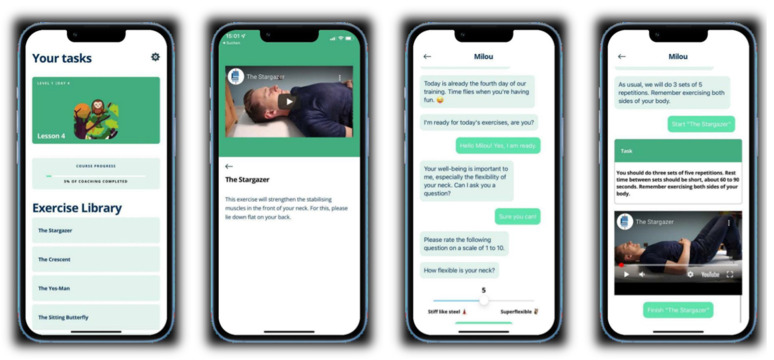
Screenshot of the ‘Milou’ smartphone application.

Patients were required to perform the exercises daily and received notifications and guidance from a chatbot. For each exercise, a video with spoken instructions was included in the app, as well as an overview of the number of series and repetitions for each exercise. The application was available for both iOS and Android operating systems. Screenshots from the application can be seen in Figure 2.

### 2.5. Outcome Measures

Most of the primary and secondary outcome measures were collected using the Tinnitus Database (https://tinnitus-database.eu) Ecological Momentary Assessment measures were collected using the smartphone application but are not reported here.

### 2.6. Primary Outcome Measure

The primary outcome measure was the 25-item Tinnitus Handicap Inventory (THI), which measures tinnitus distress by questioning the impact of tinnitus on daily living. Within the inventory, three subscales assess the functional, emotional, and catastrophic response reactions to tinnitus. The instrument has excellent internal consistency with a Cronbach’s α coefficient of 0.93 for the total scale [22]. The THI data comprised the baseline and after the 9-week study period.

### 2.7. Secondary Outcome Measure

As a secondary outcome measure, we used the German version of the Mini-TQ questionnaire [23], which is a 12-item short version of the Tinnitus Questionnaire [24]. The Tinnitus Questionnaire is a commonly used self-assessment instrument for tinnitus severity in German-speaking countries.

### 2.8. Statistics

A per-protocol analysis was performed on the study cohort. Descriptive statistics of questionnaire data were computed, and the comparability (*p* > 0.05) of both groups was analysed at the baseline using independent-samples *t*-test and chi-square tests.

A mixed model ANOVA was conducted to examine the effects of group and time point (baseline measure and final measurement after treatment) on the THI and the Mini-TQ questionnaire. The model included a random effect on the participants nested in the groups.

RStudio (R version 4.1.2) (www.r-project.org) was used for all data analysis, and a significance level of α = 0.05 was set.

## 3. Results

In total, 38 participants were initially included in the study, 19 in each group. The baseline characteristics in both groups can be found in Table 1. Both groups were comparable to each other with respect to gender distribution, age, tinnitus duration, baseline THI score, and baseline Mini-TQ score. Final assessments were received from 15 participants in the treatment group and 16 participants in the control group (see Table 2). Patients did not report unintended side effects.

### Effect on Tinnitus Distress

The results of the mixed-model ANOVA on the THI data revealed a significant interaction between group and time point (F (1, 29) = 22.53, *p* = 0.0001), indicating a reduction in the THI sum scores for the treatment group but not for the control group, during the 9-week intervention period. The main effect of the time point was F (1, 29) = 6.88, *p* = 0.0137, and the main effect of group was not significant, F (1, 29) = 0.66, *p* = 0.425. The participants in the treatment group improved from an average THI score of 51.33 (SD 23.77) at baseline to an average THI score of 38.93 (SD 21.66) at the final visit. A post hoc paired *t*-test demonstrated the strong significant decrease in the average THI score (mean difference = 12.4, t = 5.15, df = 14, *p* = 0.0001). In the same time frame, the average THI score of participants in the control group did not change significantly from 49.75 (SD 20.54) at baseline to an average THI score of 53.0 (SD 21.74) at the final assessment. In the post hoc paired *t*-test, the change in the THI scores in the control group was not statistically meaningful (mean difference = −3.25, t = −1.44, *p* = 0.17). The effect size for the interaction between group and time point was calculated using Cohen’s d. The estimate for Cohen’s d was 1.71 (95% confidence interval: [0.85; 2.56]), indicating a large effect size.

The same analysis steps were repeated for the Mini-TQ, which confirmed the results of the THI measurements. The mixed model ANOVA of the Mini-TQ data revealed a significant interaction between group and time point (F (1, 28) = 9.79, *p* = 0.0041). The main effect of the time point was significant F (1, 28) = 8.00, *p* = 0.0085), and the main effect of group allocation was not significant (F (1, 29) = 1.05, *p* = 0.314). The participants in the treatment group improved from an average Mini-TQ score of 13.27 (SD 5.81) at baseline to an average score of 9.14 (SD 5.27) at the final visit. A post hoc paired *t*-test demonstrated the strong significant decrease in the average Mini-TQ score (mean difference = 3.5, t = 3.52, df = 13, *p* = 0.0037). In the same time frame, the average Mini-TQ score of participants in the control group did not indicate a significant change from the baseline average of 13.5 (SD 5.14) to the final average of 13.56 (SD 5.68). In the post-hoc paired *t*-test, the change in the Mini-TQ scores in the control group was not statistically meaningful (mean difference = −0.06, t = −0.09, *p* = 0.93). The effect size for the difference in MiniTQ scores between the two groups was calculated using Cohen’s d. The estimate for Cohen’s d was 1.02 (95% confidence interval: [0.24; 1.80]), indicating a large effect size.

**Figure 3:**
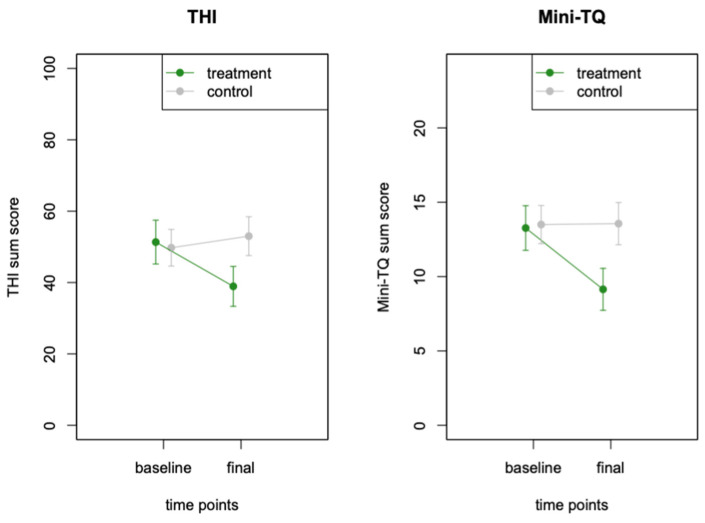
Changes in tinnitus distress for both study arms. Tinnitus distress, measured with the THI and the Mini-TQ, decreased in the treatment group but not in the control group.

Changes in the THI and Mini-TQ in both groups and at both timepoints are visualised in Figure 3.

## 4. Discussion

This study aimed to investigate the effectiveness of a smartphone application specifically directed at treating patients with ST. Our results indicate that patients in the treatment group experienced significant reductions in tinnitus distress, as measured by the Tinnitus Handicap Inventory (THI) and the Mini-Tinnitus Questionnaire (Mini-TQ), compared to the control group.

These results are similar to previous research investigating in-clinic musculoskeletal treatment for patients with ST [10,11,13,14]. The current findings confirm our clinical experience that the exercise part of the musculoskeletal treatment, in particular, is very important to obtain a good treatment outcome. Interestingly, patients appear to be able to perform these exercises at home with the guidance of a smartphone application but without the instructions of a physiotherapist. As such, these findings support the feasibility of app-based physiotherapy as a treatment option for ST, especially in areas with limited availability of musculoskeletal physiotherapists.

### 4.1. Limitations and Future Research Questions

This study is a pilot with a relatively small sample size of 31 participants, and due to the small sample size, the study has to be interpreted with caution. Despite this small sample, the observed effects were notably strong. The effect sizes for changes in the THI and Mini-TQ scores between baseline and post-treatment were large, indicating that the app-based intervention had a substantial impact on reducing tinnitus distress. However, future studies with larger sample sizes and longer follow-up periods are needed to confirm these findings.

One downside of the fixed exercise program that was included in the current smartphone application is that it is less tailored to an individual patient’s needs than a traditional in-clinic treatment would be. In the current study, it might be possible that the starting level of the exercise program was too easy for some patients or too difficult for others. Additionally, app-based treatment does not enable including manual treatment, such as manual mobilisations or trigger point deactivation techniques. From the current study, we cannot conclude whether the treatment effect in our patient sample would have been equal or larger if these patients had been treated in an in-clinic setting.

An important inclusion criterion of the study was the diagnosis of somatic tinnitus according to the Somatic Tinnitus Questionnaire—Short form (STQ-SF). Patients without a diagnosis of somatic tinnitus were excluded. This is a strength of the current study and might explain the strong effect size of the intervention. At the same time, this strict inclusion criterion also limits the generalisability of the study results. The results cannot be generalised to all subtypes of tinnitus patients. Based on this study, no conclusions can be drawn regarding patients who do not have a diagnosis of somatic tinnitus. Future replication studies need to take this inclusion criterion into consideration.

Altogether, we see three major research questions for the future: First, it will be important to compare the efficacy of app-based treatment with classic in-clinic treatment or a blended approach that combines both app-based and in-person interventions. This blended model could offer the best of both worlds—allowing for the accessibility and convenience of app-based treatment, while maintaining the hands-on guidance of in-person therapy. Second, it will be important to incorporate personalised treatment plans tailored to the individual needs and progress of each patient, which may further improve the effectiveness of the app-based intervention. Personalisation could ensure that patients are engaged in exercises that are optimally challenging and beneficial for their specific condition. Third, the results need to be replicated in a larger patient population with somatic tinnitus complaints.

### 4.2. Somatic Tinnitus Treatment via Smartphone Application

Delivering ST treatment through a smartphone application also offers several advantages over traditional clinical settings. One key benefit is increased accessibility, as patients can access the treatment anytime and anywhere. This flexibility reduces the need for frequent clinic visits, making the treatment more feasible for individuals with mobility issues or those living in remote areas. Additionally, the app provides convenience by allowing patients to perform exercises at their own pace and according to their schedules. This flexibility may enhance adherence to the treatment regimen, addressing a common barrier seen in traditional physiotherapy where patients may struggle to maintain compliance with home exercises.

From a cost perspective, using a smartphone app lowers the expenses associated with in-person therapy sessions, including travel costs and potential time off work. Furthermore, app-based interventions offer scalability, enabling the treatment to reach a larger population without the constraints of clinical capacity. This could allow many more individuals to benefit from treatment who may otherwise be unable to access physiotherapy for somatic tinnitus.

Finally, the app-based treatment enables continuous data collection on patient progress and adherence, offering valuable insights for both patients and clinicians. This real-time data can be used to monitor treatment effectiveness and make necessary adjustments, improving the overall quality of care. This feedback loop between patient performance and app functionality has the potential to significantly enhance patient outcomes and treatment satisfaction.

## 5. Conclusions

In conclusion, this pilot study provides evidence that an app-based physiotherapy intervention could be an effective option for treating ST. While further research is necessary to confirm these results in a larger sample and to compare its efficacy with traditional and blended treatment models, the potential advantages in terms of accessibility, cost, and personalisation make digital treatment delivery a promising option for the future management of ST.

## Figures and Tables

**Table 1:** Baseline characteristics in both groups. For age, tinnitus duration, and baseline questionnaire scores, we report mean and standard deviation (SD) in brackets.

Characteristic	Treatment Group	Control Group	*p*-Value
Sample Size: *N*	15	16	
Gender: f/m	9/6	7/9	0.586
Age: years (std)	50.93 (12.78)	48.00 (13.60)	0.542
Tinnitus Duration: months (std)	90.00 (84.71)	94.85 (94.50)	0.892
Baseline THI Score: mean (std)	51.33 (23.77)	49.75 (20.54)	0.844
Baseline Mini-TQ Score: mean (std)	13.27 (5.81)	13.5 (5.13)	0.907

**Table 2:** Clinical parameters at baseline and final visit. For both questionnaires, THI and Mini-TQ, we show the mean values and standard deviations in brackets. The results of the mixed-model ANOVA, examining the interaction effect, are presented, including the corresponding F-value and *p*-value. Cohen’s is presented together with the 95% confidence interval.

Parameter	Treatment Group	Control Group	F	*p*	Cohen’s d [95% CI]
THI Score					
Baseline	51.33 (23.77)	49.75 (20.54)			1.71
Final	38.93 (21.66)	53.0 (21.74)	F (1, 29) = 22.53	0.0001	[0.85; 2.56]
Mini-TQ Score					
Baseline	13.27 (5.81)	13.5 (5.14)			1.02
Final	9.14 (5.27)	13.56 (5.68)	F (1, 28) = 9.79	0.0041	[0.24; 1.80]

## Data Availability

Requests to access the dataset can be directed to W.S.
